# Endothelial Dysfunction and Impaired Neurovascular Coupling Responses Precede Cognitive Impairment in a Mouse Model of Geriatric Sepsis

**DOI:** 10.3389/fnagi.2021.644733

**Published:** 2021-05-14

**Authors:** Tamas Csipo, Benjamin R. Cassidy, Priya Balasubramanian, Douglas A. Drevets, Zoltan I. Ungvari, Andriy Yabluchanskiy

**Affiliations:** ^1^Center for Geroscience and Healthy Brain Aging, Department of Biochemistry and Molecular Biology, University of Oklahoma Health Sciences Center, Oklahoma City, OK, United States; ^2^International Training Program in Geroscience, Department of Public Health, Doctoral School of Basic and Translational Medicine, Semmelweis University, Budapest, Hungary; ^3^Department of Medicine, University of Oklahoma Health Sciences Center, Oklahoma City, OK, United States; ^4^Department of Veterans Affairs Medical Center, Oklahoma City, OK, United States; ^5^International Training Program in Geroscience, Departments of Medical Physics and Informatics, Theoretical Medicine Doctoral School University of Szeged, Szeged, Hungary

**Keywords:** aging, sepsis, neurovascular coupling, endothelial dysfunction, cognition

## Abstract

Sepsis is a life-threatening condition, the incidence of which is significantly increased in elderly patients. One of the long-lasting effects of sepsis is cognitive impairment defined as a new deficit or exacerbation of preexisting deficits in global cognition or executive function. Normal brain function is dependent on moment-to-moment adjustment of cerebral blood flow to match the increased demands of active brain regions. This homeostatic mechanism, termed neurovascular coupling (NVC, also known as functional hyperemia), is critically dependent on the production of vasodilator NO by microvascular endothelial cells in response to mediators released from activated astrocytes. The goal of this study was to test the hypothesis that sepsis in aging leads to impairment of NVC responses early after treatment and that this neurovascular dysfunction associates with impairments in cognitive performance and vascular endothelial dysfunction. To test this hypothesis, we used a commonly studied bacterial pathogen, *Listeria monocytogenes*, to induce sepsis in experimental animals (males, 24 months of age) and subjected experimental animals to a standard clinical protocol of 3 doses of ampicillin i.p. and 14 days of amoxicillin added to the drinking water. NVC responses, endothelial function and cognitive performance were measured in septic and age-matched control groups within 14 days after the final antibiotic treatment. Our data demonstrate that sepsis in aging significantly impairs NVC responses measured in somatosensory cortex during whisker stimulation, significantly impairs endothelial function in isolated and pressure cannulated aorta rings in response to acetylcholine stimulation. No significant impairment of cognitive function in post-sepsis aged animals has been observed when measured using the PhenoTyper homecage based system. Our findings suggest that sepsis-associated endothelial dysfunction and impairment of NVC responses may contribute to long-term cognitive deficits in older sepsis survivors.

## Introduction

Sepsis is a life-threatening condition, the incidence of which is significantly increased and the outcomes are dramatically worsened in elderly patients (Angus et al., 2001; Martin et al., 2003; Lagu et al., 2012; Corrales-Medina et al., 2015). Older adults (>65 years of age) are 10–15 times more likely to be hospitalized with sepsis than younger individuals and over 60% of older adults admitted to the intensive care unit present with sepsis upon admission (Marik, 2006). Severe sepsis accounts for up to half of bed-days in older adults (Wood and Angus, 2004; Hall et al., 2011). The incidence of sepsis in older adults is likely even higher than reported because many elderly dying patients with infection are not documented as having “sepsis” as they often receive palliative care rather than being sent to the ICU for aggressive treatment (Starr and Saito, 2014). Thus, sepsis in elderly patients is one of the most expensive conditions treated in the US hospitals, the costs for which are exceeding $60 billion per year (Andrews and Elixhauser, 2006; Wier and Andrews, 2006).

The hallmark of sepsis is exaggerated systemic inflammatory response to infection, the consequences of which include generalized microvascular dysfunction, impaired tissue perfusion, endothelial activation and coagulation abnormalities (Hotchkiss et al., 2016). It is now established that a number of sepsis survivors experience residual physical and psychological effects long after treatment and discharge from the hospital (Iwashyna et al., 2010; Calsavara et al., 2018a). One of the long-lasting effects of sepsis is cognitive impairment defined as a new deficit or exacerbation of preexisting deficits in global cognition or executive function (Calsavara et al., 2018a). Clinical data demonstrate that nearly 35% of elderly develop post-sepsis cognitive deficits in the magnitude comparable to individuals with moderate traumatic brain injury or mild Alzheimer’s disease (Pandharipande et al., 2013).

Normal brain function is critically dependent on moment-to-moment adjustment of cerebral blood flow to match the increased demands of active brain regions (Toth et al., 2014, 2015a,2015b; Tucsek et al., 2014; Tarantini et al., 2015). This homeostatic mechanism, termed neurovascular coupling (NVC, also known as functional hyperemia), depends on the production of vasodilator NO by microvascular endothelial cells in response to mediators released from activated astrocytes. Extensive clinical and pre-clinical data demonstrate that NVC is impaired in aging (Csiszar et al., 2019; Farias Quipildor et al., 2019; Tarantini et al., 2019b) due, at least in part, to cerebromicrovascular endothelial dysfunction and is causally linked to cognitive impairment (Toth et al., 2014; Tarantini et al., 2015; Ungvari et al., 2017). Further, there is emerging evidence from experimental studies that restoration of NVC responses in aged animals leads to improved cognitive performance (Tarantini et al., 2018, 2019a; Csipo et al., 2019b; Lipecz et al., 2019). There is strong evidence that sepsis is associated with significant endothelial dysfunction, which contributes to the pathogenesis of multiple organ failure in severe cases (Aird, 2003; Peters et al., 2003; Szabo and Goldstein, 2011; Vachharajani et al., 2012; Coletta et al., 2014; De Backer et al., 2014). Aging is associated with impaired endothelial stress resilience (Ungvari et al., 2011a, b; Fulop et al., 2018) and there are strong experimental data suggesting that sepsis in aged mice is associated with exacerbated endothelial dysfunction (Coletta et al., 2014). However, the impact of sepsis on NVC responses in aging is currently unknown.

This study was designed to test the hypothesis that sepsis in aging leads to impairment of NVC responses early after treatment and that this neurovascular dysfunction associates with impairments in cognitive performance and vascular endothelial dysfunction. To test this hypothesis, we used a commonly studied bacterial pathogen, *Listeria monocytogenes*, to induce sepsis in experimental animals. In humans, *L. monocytogenes* is a food-borne pathogen known to primarily affect the elderly population in whom it causes sepsis and central nervous system infections (Yildiz et al., 2007). For example, adults >65 years of age are over 4 times more likely than the general population to experience invasive *L. monocytogenes* infections, and patients with *L. monocytogenes* CNS infections typically have worse outcomes than those infected with other common neuroinvasive bacteria (Tubiana et al., 2020). To mimic the clinical scenario, antibiotic treatment was administered. Cognitive performance, NVC responses and vascular endothelial function were measured 30 days after sepsis induction and within 2 weeks after antibiotic treatment completion.

## Materials and Methods

### Sepsis Model and Experimental Animals

*L. monocytogenes* was used to induce sepsis in experimental animals. *L. monocytogenes* strain EGD was originally obtained from P.A. Campbell (Dramsi et al., 1996). Bacteria were stored in brain-heart infusion (BHI) broth (Difco, Detroit, MI) at 109 CFU/mL at −80°C. For experiments, the stock culture was diluted 1:10,000 into BHI and cultured overnight at 37°C with shaking.

This study was carried out with the approval from Institutional Animal Care and use Committee (IACUC) of the University of Oklahoma HSC (OUHSC). All animals were obtained from the NIA Aged Rodent Colony (Charles River Laboratories, Wilmington, MA).

Male C57BL/6N mice 24 months of age were used in experiments as indicated. The biological age of 24 months old mice corresponds to that of 65 year old humans. Mice were injected i.p. with 500–1,000 μL PBS containing 2.0–7.5 × 104 CFU *L. monocytogenes* (sepsis group, *n* = 27) or vehicle (sham group, *n* = 11), and then treated with antibiotics as previously described (Zhang et al., 2018). Although *L. monocytogenes* is a foodborne bacterial pathogen in humans, the choice for i.p. administration in our experimental design was based on amino acid difference between human and murine E cadherin. This difference significantly limits the invasion of *L. monocytogenes* through the GI in mice (Bonazzi et al., 2009), therefore the i.v. or i.p. injections are commonly used to bypass the GI tract in mice and to establish systemic infection in a reproducible fashion (Zhang et al., 2018; Cassidy et al., 2020).

Septic and sham mice were injected i.p. with 2 mg ampicillin (Butler Schein Animal Health, Dublin, OH) three times at 10–12 h intervals beginning 48 h post-infection. Bubblegum-flavored amoxicillin (2 mg/mL final concentration) was added to the drinking water 3 days post-infection and continued until sacrifice or 14 days post-infection. All solutions were warmed to body temperature prior to injection. Mice were weighed daily for 14 days. Mice were euthanized by CO_2_ asphyxiation and transcardially perfused with ice cold PBS.

### Measurement of Neurovascular Coupling Responses

Mice in each group were anesthetized with isoflurane (4% induction for 1–2 min, 1% maintenance during surgical preparation, and 0.5% during NVC measurements), endotracheally intubated and ventilated (MousVent G500; Kent Scientific Co., Torrington, CT) as described previously (Tarantini et al., 2017). A thermostatic heating pad (Kent Scientific Co., Torrington, CT) was used to maintain rectal temperature at 37°C. End-tidal CO_2_ was controlled between 3.2 and 3.7% to keep blood gas values within the physiological range. Cannulation of the right femoral artery was performed for arterial blood pressure measurement (Living Systems Instrumentations, Burlington, VT). The blood pressure was within the physiological range throughout the experiments (90–110 mmHg). Mice were immobilized and placed on a stereotaxic frame (Leica Microsystems, Buffalo Grove, IL), the scalp and periosteum were pulled aside, and the skull was gently thinned using a dental drill while cooled with dripping buffer.

Prior to assessment of NVC responses, experimental animals were maintained on 0.5% isoflurane for 20 min. To assess NVC, a laser speckle contrast imager (Perimed, Järfälla, Sweden) was placed 10 cm above the thinned skull and differential perfusion maps of the brain surface were captured (Tarantini et al., 2017). Changes in cerebral blood flow were monitored above the left and right whisker barrel cortex in six consecutive and alternating trials of left and right whisker stimulation, separated by 5–10 min intervals.

For data evaluation, the relative change in the CBF signal was compared between the baseline of the region of interest (ROI) and during stimulation. To rule out an unspecific increase of cerebral blood flow, the difference between the relative change for both lateral ROI‘s was used for further evaluation to determine specific changes for the contralateral somatosensory cortex. In each study, the experimenter was blinded to the treatment of the animals.

### Assessment of Endothelial Nitric Oxide (NO)-Mediated Vasodilation in Aorta

Assessment of endothelium-dependent vasorelaxation was performed in isolated aorta ring preparations as previously described (Horvath et al., 2005; Csiszar et al., 2008; Pearson et al., 2008; Csiszar et al., 2009; Bailey-Downs et al., 2012). In brief, vessels were cut into ring segments approximately 1.5 mm in length and were mounted in wire myograph chambers (Danish Myo Technology A/S, Inc., Denmark) for measurement of isometric tension. The chambers were filled with Krebs buffer solution (118 mM NaCl, 4.7 mM KCl, 1.5 mM CaCl_2_, 25 mM NaHCO_3_, 1.1 mM MgSO_4_, 1.2 mM KH_2_PO_4_, and 5.6 mM glucose; at 37°C; gassed with 95% O_2_ and 5% CO_2_). Optimal passive tension was applied to the rings (as determined from the vascular length-tension relationship). After 1 h equilibration, rings were pre-constricted with 10 μM phenylephrine. Acetylcholine-mediated vasodilation was quantified as a normalized relaxation compared to phenylephrine preconstriction. Endothelial NO-dependent relaxation was measured in the presence and absence of the NO synthase inhibitor N(ω)-nitro-L-arginine methyl ester in response to acetylcholine (from 1 nM up to 10 μM). Endothelium independent relaxation was determined in response to sodium nitroprusside (SNP, 0.1 nM to 10 μM).

### Assessment of Cognitive Function Using the PhenoTyper Home Cage System

Cognitive performance was tested using the automated home-cage testing apparatus, PhenoTyper (Model 3000, Noldus Information Technology, Netherlands), as described previously (Logan et al., 2019). Mice were continuously monitored during the light and dark cycles. During cognitive testing, mice were rewarded with a food pellet after five successful entries into the correct hole (FR5; fixed ratio 5) as previously described (Logan et al., 2018). In brief, the percentage of correct entries made in a moving window of the trailing 30 entries was calculated and trials to reach an 80% success rate were determined. Data was tracked for the dark and light phases over the course of 4 days. For initial learning phase, mice were required to pass through the left entrance of the CognitionWall to obtain a food reward (Dustless Precision Rodent Pellets, F05684, Bio-Serv, Flemington, NJ), which was dispensed using an FR5 schedule. In 48 hours, the task was modified, and the correct response was changed to the right entry requiring the animal to extinguish the previous learning and acquire a new response, and the cognitive flexibility was calculated. The data were processed using Python programming language. The Leaning Index for initial learning and cognitive flexibility phases was calculated as correct entries minus the incorrect entries divided by the total number of entries.

### Statistical Analysis

Mortality rates were analyzed using Chi-square test and data were presented in the form of Kaplan-Meier curves. NVC responses were evaluated using the appropriate unpaired *t*-test after testing the dataset for normality. Relaxation of aorta rings were expressed as the change of ring tension relative to the tension evoked by the preconstriction. Raw tension data of rings not exhibiting a constriction greater than 1 mN and/or endothelium-dependent vasorelaxation were excluded from further analysis. Multiple aorta ring measurements were then averaged for each animal, and two-way repeated measures ANOVA with Sidak’s *post hoc* test was used to compare relaxation between groups. Cognitive data from the PhenoTyper cages was calculated and presented as changes in initial learning during the first 2 days of cognitive assessment, and as cognitive flexibility during the reversal phase of cognitive assessment. Initial learning and cognitive flexibility were calculated as a number of ([correct entries-incorrect entries]/total entries) during the hours 3–12 and 51–62, respectively. Cognitive data were analyzed using the parametric unpaired *t-*test after passing the normality test. All statistical analysis was performed in GraphPad Prism 8.4.2 (GraphPad Software, LLC, United States). Data are shown as mean ± SEM. A *p* < 0.05 was considered statistically significant.

## Results

### The Effect of *L. monocytogenes* Induced Sepsis on Mortality Rates and Body Weights in Aged Mice

**FIGURE 1:**
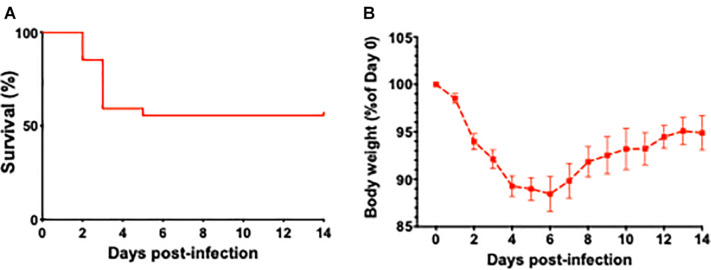
The effect of *L. monocytogenes* induced sepsis on mortality rates and body weights in aged mice. **(A)** Survival curve of 24-month-old mice after *L. monocytogenes* infection and antibiotic treatment. Over the course of the experiment, 13 out of 27 *L. monocytogenes* infected mice died due to sepsis. Antibiotic treatment was initiated 48 h post-infection. **(B)** Time course of changes in body weight associated with *L. monocytogenes* induced sepsis in aged mice.

Survival curves of aged mice after infection with *L. monocytogenes* are shown in Figure 1A. The weight loss is considered a key biological feature of sepsis in mouse models (Stortz et al., 2019) and the time course of changes in body weight associated with *L. monocytogenes* induced sepsis is shown in Figure 1B. The weight loss in current project was similar to reported data that previously utilized similar sepsis model (Cassidy et al., 2020). The timescale of observed mortality and weight loss in our sepsis model was also similar to sepsis timescale in humans, when the vast majority of deaths in first 72 h after admission to the ICU are due to primary infection-related multiple organ failure (Daviaud et al., 2015).

One mouse from the experimental group was euthanized per recommendations of attending veterinarian for conditions not associated with experimental sepsis protocol and were removed from analysis.

### Sepsis Impairs NVC Responses in Aged Animals

**FIGURE 2:**
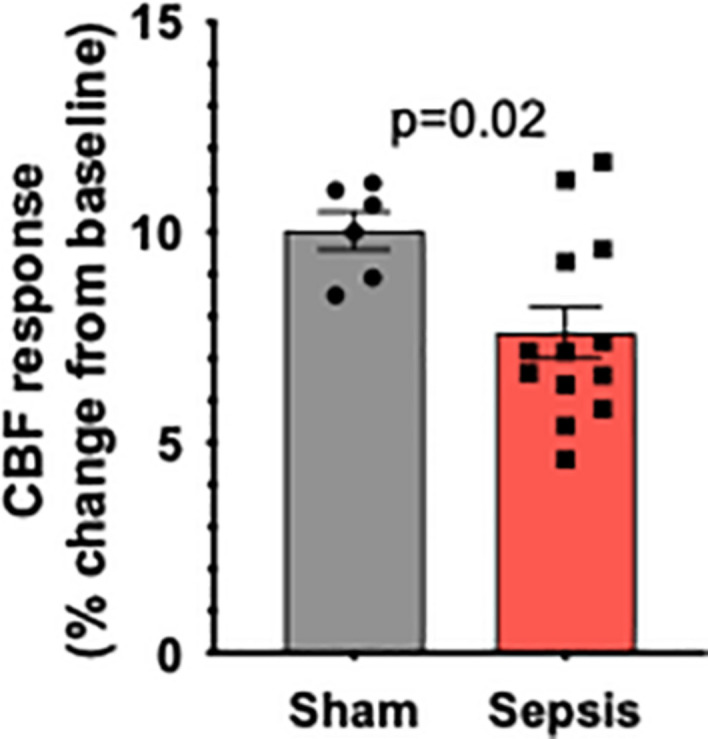
Sepsis impairs NVC responses in aged animals. Sepsis (*n* = 13) significantly impaired CBF responses during whisker stimulation when compared to sham controls (*n* = 7). Data are presented as a%-change from baseline values prior to sham or *l.* monocytogenes treatment. *p* = 0.002.

To determine the effect of sepsis on NVC responses in aging, we assessed functional hyperemia in the whisker barrel cortex in mice 5 days after completion of antibiotic treatment in infected and sham-treated aged animals (*n* = 13 and *n* = 7, respectively). One mouse in sham group died due to complications with the surgical preparation and their aortas were immediately removed for vascular functional assessment. We found that in aged mice sepsis significantly impaired NVC responses evoked by contralateral whisker stimulation (Figure 2). Our data demonstrate that sepsis-induced chronic neurovascular dysfunction persists even after a complete course of translationally relevant antibiotic treatment.

### Sepsis Impairs Vascular Endothelial Function in Aged Animals

**FIGURE 3:**
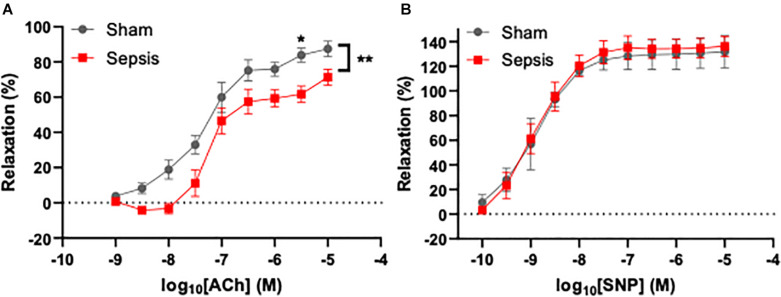
Sepsis caused by *L. monocytogenes* was associated with endothelial dysfunction. Endothelium-dependent vasodilation was measured using wire myography in aorta rings under isometric conditions. **(A)** Sepsis significantly reduced endothelial function in aged animals. A significant difference in dilation between groups was observed when 4 μM acetylcholine (ACh) was applied to aorta rings. **(B)** Endothelium-independent relaxation evoked by sodium nitroprusside (SNP) was not different between groups (right panel). *p < 0.05; **p < 0.01.

To assess the effect of *L. monocytogenes* induced sepsis on endothelial function, acetylcholine-mediated relaxation was measured in aorta rings under isometric conditions (*n* = 13 for the septic group, *n* = 7 for sham). Acetylcholine-induced relaxation was compared with two-way repeated measures ANOVA (Figure 3A), which showed that sepsis accounted for 5.122% of variance [*F*_(1,14)_ = 9.64, *p* = 0.008]. *Post hoc* multiple Sidak’s test showed a significant difference between groups at acetylcholine concentration of 4 μM (mean difference: 22.12%, 95% CI: 1.51–42.72, *p* = 0.03). Inhibition of endothelial NO synthase abolished acetylcholine-evoked relaxation in both groups (data not shown). Endothelium independent vasorelaxation in response to sodium nitroprusside was not different between the groups (Figure 3B). These data suggest that sepsis induces endothelial dysfunction, which persists even after completion of a full antibiotic treatment.

### Sepsis Effects on Cognitive Performance in Aged Animals

**FIGURE 4:**
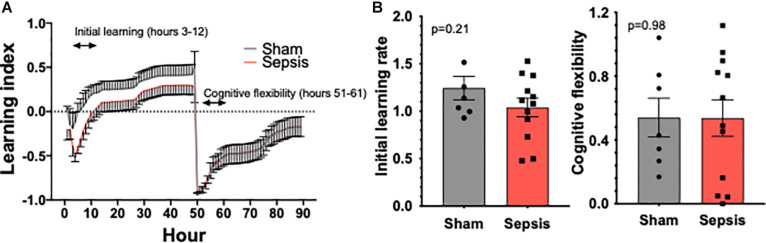
Sepsis effects on cognitive performance in aged animals. PhenoTyper homecage system was used to evaluate the impact of sepsis on cognitive performance (*n* = 13 in sepsis group and *n* = 7 in sham group). **(A)** All experimental animals were tested for the duration of 4 days, during which Initial Learning and Cognitive Flexibility were evaluated. Our data demonstrate a trend toward an impairment of cognitive performance measured during the initial learning phase **(B)**. Cognitive flexibility was unaltered by sepsis.

To evaluate whether *L. monocytogenes* induced sepsis has an impact on cognition, experimental animals were evaluated for cognitive performance using the PhenoTyper homecage system (*n* = 13 in sepsis group and *n* = 7 in sham group, Figure 4A). One mouse in sepsis group was excluded from the analysis due failure in food reward/pellet dispensing system. No significant effect on cognitive performance measured using the PhenoTyper homecage system in *L. monocytogenes* induced sepsis in this model was observed (Figure 4B).

## Discussion

In this study, we utilized a clinically relevant *in vivo* model of sepsis via infection of aged mice with *L. monocytogenes*, an important cause of bacterial sepsis in older adults (Yildiz et al., 2007). The key finding of this study is that in aged mice, *L. monocytogenes* induced sepsis impairs NVC responses and promotes endothelial dysfunction, a finding which persists even after treatment with a clinically relevant regimen of antibiotics.

This is the first study to demonstrate compromised NVC responses in a relevant preclinical animal model of geriatric sepsis. Previous clinical studies reported multifaceted effects of sepsis on cerebral blood flow regulation (Terborg et al., 2001; Bowie et al., 2003; Pfister et al., 2008; Toksvang et al., 2014; Vasko et al., 2014; de Azevedo et al., 2017; Crippa et al., 2018; Masse et al., 2018). The emerging concept is that both reduced cerebral blood flow and altered autoregulation contribute to sepsis-associated encephalopathy. Our data are in line with this concept, suggesting that impaired moment-to-moment regulation of cerebral blood flow is casually linked to the pathogenesis of cognitive impairment associated with geriatric sepsis. There is a temporal evolution of cerebral hemodynamic impairments in sepsis. Our data suggest that persisting neurovascular dysfunction is manifested even after adequate antibiotic treatment in aged mice.

An abundant body of evidence demonstrates that sepsis due to a wide range of bacterial infections results in generalized endothelial dysfunction (Szabo and Goldstein, 2011; Becker et al., 2012; Wexler et al., 2012). Here we provide evidence that *L. monocytogenes* infection also causes marked endothelial dysfunction, which persists after antibiotic treatment. Various bacterial species can exert either a direct effect on the vascular endothelium or promote endothelial dysfunction through the induction of pro-inflammatory mediators and oxidative stress (Al-Obaidi and Desa, 2018). *L. monocytogenes* is known to release the pore-forming toxin listeriolysin O, which confers deleterious effects on endothelial cells (Drevets, 1998; Wilson and Drevets, 1998; Kayal et al., 1999; Kayal et al., 2002). Additionally, *L. monocytogenes* can also invade brain microvascular endothelial cells (Drevets et al., 1995; Greiffenberg et al., 1998). Our findings that *L. monocytogenes* infection promotes significant endothelial dysfunction in aged mice is translationally relevant, as healthy endothelial function is a prerequisite for normal NVC responses. The limitation to our findings is that endothelial function measured in aorta rings cannot be directly translated to the function of endothelial cells in the cerebral microcirculation due to known significant morphological and functional differences between endothelial cells from different anatomic sites (Sengoelge et al., 2005). However, recent studies suggest that the extent of generalized endothelial dysfunction predicts cognitive performance in older adults (Csipo et al., 2019a), providing additional evidence on the role of endothelial dysfunction in impairment of NVC responses in sepsis. Further, while not the part of this study, future experiments are warranted to evaluate the contribution of endothelial function in hemodynamic responses of NVC measured *in vivo* (such as in L-NAME and acetylcholine infused animals).

A growing body of clinical evidence demonstrates that persisting cognitive impairment is one of the major complications observed in sepsis survivors (Iwashyna et al., 2010). Recent meta-analysis studies report that cognitive impairment is observed in up to 21% of sepsis survivors and that sepsis may affect different domains of cognition including attention, cognitive flexibility, processing speed and working memory (Calsavara et al., 2018b). The study reports that cognitive decline can be observed as early as 24 h after discharge from the intensive care unit in young and middle-aged sepsis survivors of 34–64 years of age. When tested 1 year after sepsis incident, all subjects presented with a general improvement in cognitive performance for Verbal Fluency, Mini Mental State Examination, Word Listing Learning, Word List Recall, Word List Recognition and Praxis Recall. Interestingly, cognitive performance based on Modified Boston Naming Test and Constructional Praxis remained unchanged from the time of discharge from intensive care unit, suggesting sepsis has profound and long-lasting effects on cognitive performance in young and middle-aged adults (Calsavara et al., 2018b). Further, clinical studies report that cognitive impairment among sepsis survivors of > 65 years of age is significantly associated with substantial and persistent new cognitive impairments and functional disability 1-year post-sepsis, with the magnitude of these deficits likely leading to loss of independence and severely reduced quality of life. In our study, we did not observe significant difference in cognitive performance early post-sepsis when compared to control animals. Possible explanation for non-significant difference in cognitive performance, but just a trend, could be due to the sample size and due to the choice of behavioral test. PhenoTyper homecage system that was used in our experiments is the food reward-based spatial memory task (Logan et al., 2018), and while it evaluates hippocampal function, it does not allow evaluation of other domains of cognitive function that are known to be most affected in sepsis in humans (Calsavara et al., 2018b). Therefore, whether cognitive deficits observed in the model used in the current study are present in other domains of cognition and whether the relationship between impairment of NVC responses and cognitive dysfunction exist is yet to be studied.

## Conclusion

In conclusion, findings from our study suggest that sepsis-associated endothelial dysfunction and impairment of NVC responses in aged animals may contribute to long-term cognitive deficits in older sepsis survivors. Future studies are warranted to determine whether pharmacological interventions aimed at restoration of endothelial function and NVC responses are effective in improving cognitive function in sepsis survivors.

## Data Availability Statement

The raw data supporting the conclusions of this article will be made available by the authors, without undue reservation.

## Ethics Statement

The animal study was reviewed and approved by The Institutional Animal Care and Use Committee at the University of Oklahoma Health Sciences Center, Protocol #17-072-SSL.

## Author Contributions

TC, BC, DD, ZU, and AY designed and performed the study. All authors contributed to the article and approved the submitted version.

## Conflict of Interest

The authors declare that the research was conducted in the absence of any commercial or financial relationships that could be construed as a potential conflict of interest.
